# Temporal dynamics of amygdala response to emotion- and action-relevance

**DOI:** 10.1038/s41598-020-67862-1

**Published:** 2020-07-07

**Authors:** Raphael Guex, Constantino Méndez-Bértolo, Stephan Moratti, Bryan A. Strange, Laurent Spinelli, Ryan J. Murray, David Sander, Margitta Seeck, Patrik Vuilleumier, Judith Domínguez-Borràs

**Affiliations:** 1 Laboratory for Behavioral Neurology and Imaging of Cognition, Campus Biotech, University of Geneva, Geneva, Switzerland; 2 Pre-surgical Epilepsy Evaluation Unit, Clinic of Neurology, University Hospital, Geneva, Switzerland; 3 Swiss Center for Affective Sciences, University of Geneva, Geneva, Switzerland; 4 Department of Clinical Neurosciences, University of Geneva, Geneva, Switzerland; 5 Facultad de Psicología, Universidad Autónoma de Madrid, Madrid, Spain; 6 Department of Experimental Psychology, Complutense University of Madrid, Madrid, Spain; 7 Laboratory for Clinical Neuroscience, Centre for Biomedical Technology, Universidad Politécnica de Madrid, Madrid, Spain; 8 Department of Neuroimaging, Alzheimer’s Disease Research Centre, Reina Sofia-CIEN Foundation, Madrid, Spain; 9 Laboratory for the Study of Emotion Elicitation and Expression, Department of Psychology, University of Geneva, Geneva, Switzerland; 10 Laboratory for Behavioral Neurology and Imaging of Cognition, Department of Neuroscience, University Medical Center, 1 Rue Michel-Servet, 1211 Geneva, Switzerland; 11 Present Address: Department of Clinical Psychology and Psychobiology, University of Barcelona, Barcelona, Spain

**Keywords:** Amygdala, Neurophysiology

## Abstract

It has been proposed that the human amygdala may not only encode the emotional value of sensory events, but more generally mediate the appraisal of their relevance for the individual’s goals, including relevance for action or task-based needs. However, emotional and non-emotional/action-relevance might drive amygdala activity through distinct neural signals, and the relative timing of both kinds of responses remains undetermined. Here, we recorded intracranial event-related potentials from nine amygdalae of patients undergoing epilepsy surgery, while they performed variants of a Go/NoGo task with faces and abstract shapes, where emotion- and action-relevance were orthogonally manipulated. Our results revealed early amygdala responses to emotion facial expressions starting ~ 130 ms after stimulus-onset. Importantly, the amygdala responded to action-relevance not only with face stimuli but also with abstract shapes (squares), and these relevance effects consistently occurred in later time-windows (starting ~ 220 ms) for both faces and squares. A similar dissociation was observed in gamma activity. Furthermore, whereas emotional responses habituated over time, the action-relevance effect increased during the course of the experiment, suggesting progressive learning based on the task needs. Our results support the hypothesis that the human amygdala mediates a broader relevance appraisal function, with the processing of emotion-relevance preceding temporally that of action-relevance.

## Introduction

The amygdala is a crucial component of brain circuits allowing swift reaction to threatening stimuli, an ability critical for adaptive behavior and survival^1,2^. Fast and efficient discrimination of potentially harmful events is a hallmark of the fear response, associated with a well-established sensitivity of the amygdala to threat information, and extensive connectivity with multiple other brain regions that act to facilitate attention, enhance memory, and promote actions^3^.

It has recently been questioned, however, whether the amygdala is dedicated to fear processing^1^ or whether instead it may serve a broader function for the appraisal of behaviorally relevant events (see^4^ for a review). There is abundant evidence that the human amygdala responds to other emotionally significant stimuli beyond threat^5^, including positive or reward information^6,7^, but also novelty^8^ and non-emotional salient stimuli with personal impact or goal-related significance^9,10^. This diversity of response patterns has led to recent theoretical accounts proposing that the amygdala may actually encode the “relevance” of events, which is determined by the goals, needs, or values of the individual, in a context-dependent manner^11^. This account accords with psychological theories, such as the component process model of emotional appraisal, put forward by Scherer^12^ and Sander et al.^13^. In this framework, refers to any information or event that directly implies the achievement or obstruction of an observer’s physical, motivational (i.e. emotional), and/or task-based (i.e. action-related) goal, need, or desire^4,14,15^. This theoretical account postulates that the detection of relevance of an event is a primary step in the elicitation of any emotion^12^ and can thus initiate a cascade of other cognitive appraisals. Moreover, in this view, the intrinsic affective value of an event and the current goals of an individual may not only interact to shape the nature of elicited emotions^12,16^, but also jointly impact on patterns of motor expression and behavioral actions evoked by the situation^12^. Likewise, other views suggest that emotional signals (and associated stimulus processing in amygdala and connected regions) are intimately related to the encoding of behavioral goal values^17^. This may occur both in the long-term perspective of biological needs, or in a more short-term perspective, conceptualized as information relevant to adjust behavior in order to perform a current task^9,10^.

Indirect support to such relevance hypothesis has come from several functional magnetic resonance imaging (fMRI) studies using emotional stimuli, as well as non-emotional stimuli. These studies reported that the amygdala is implicated in the detection of non-emotional features such as stimulus saliency^18^, stimulus intensity^19^, or experiential arousal^20^, as well as in the assignment of stimulus value^21^ or the encoding of goal-value^22^. Other studies found that amygdala activity is modulated in conditions where stimulus relevance is experimentally manipulated by the current task design. On one hand, studies using emotional stimuli, such as scenes with affective content^20,21^, emotional words^23^, or emotional faces^24^, showed greater emotion-related activity in the amygdala when the stimuli were also task- or action-relevant (i.e. targets). However, recent meta-analyses indicate strong amygdala responses even during passive viewing conditions of emotional stimuli^25^. On the other hand, several studies using non-emotional stimuli, such as letters or numbers previously associated with a particular task-dependent value, showed robust activation within the amygdala unrelated to any affective value^26,27^. This would accord with the relevance hypothesis postulating that a primary role of the amygdala is to compute the personal significance of an event, in relation to current goals and needs, so as to motivate adaptive actions and behaviors^4^.

In this theoretical framework, a representative and simplified form of goal-related values, other than affective or motivational values (i.e., those directly related to survival or homeostatic processes), is action-relevance. Action-relevance can be considered as the significance of an event summoning action for the pursuit of immediate goals, such as reacting to a target stimulus in a task. However, it remains unclear whether and how the human amygdala encodes action-relevance, regardless of any emotion-related value. Flexible, task-dependent neuronal responses in amygdala to current behaviorally relevant information might occur in the same fast and rapid manner as responses to intrinsic emotional relevance such as threat^28–30^, or rather arise through slower processing of cognitive information, encoded in higher-level cortical areas associated with attention and executive task control^10,29,31^. Hence, a direct comparison of the processing of emotional and non-emotional relevance signals (such as action-relevance) in the amygdala is critical in order to better understand its function and assess any selectivity to particular emotions. However, previous fMRI studies cannot shed light on this question since BOLD activity lacks the temporal resolution necessary to determine the exact dynamics underlying these processes.

In the current study, we therefore leveraged direct recordings of amygdala activity with millisecond resolution, by investigating patients who underwent intracranial electroencephalography (iEEG) for epilepsy surgery. This allowed us to directly compare neural responses to visual stimuli associated with different emotional value and different task goals. We used a modified version of a Go/NoGo paradigm^32^ to examine the effect of task-relevance, operationalized here through motor action. Recent fMRI studies in healthy participants reported greater amygdala responses to target, relative to non-target, emotional stimuli^33^, but also selective increases to non-emotional *Go* events relative to *NoGo* events^26^. Conversely, amygdala responses to emotional faces are reduced on *NoGo* trials^24^. Although these findings would accord with the hypothesis of action-relevance, the temporal dynamics of such response patterns cannot precisely be established with fMRI. Studies using scalp EEG in healthy participants reported that brain responses to target and non-target emotional stimuli in classical Go/NoGo tasks differ around 300 ms^34^ over frontocentral areas (FC1, Cz), a latency that is much delayed in comparison with early amygdala activation to emotionally relevant stimuli observed with iEEG (e.g., typically 100–200 ms post-onset; see^35^). In addition, a recent study using iEEG^10^ observed different populations of neurons in medial temporal lobe that responded to either targets or distractors during a visual search task, but a manual response was required for both kinds of stimuli, and these neurons were equally found in amygdala and hippocampus. Here, by exploiting both the anatomical and temporal resolution of iEEG, we sought to directly compare the encoding of both action-based and emotion-based relevance within the amygdala using the same category of stimuli, across a comprehensive time-window, and to test for their potential functional interplay. Further, given previous evidence that amygdala activity may evolve over time due to novelty^36^, learning^37^, or habituation^38^, we also compared these two types of relevance effects during the initial and final parts of our tasks.

## Materials and methods

### Participants

Seven epileptic patients (4 females) participated in the study prior to brain surgery for pharmacologically intractable epilepsy (see Table 1 for demographic details). The epileptic focus was outside the amygdala for all patients, ensuring reliable recordings from healthy brain tissues. Written informed consent was obtained for each patient, all methods used in this study were performed in accordance with the relevant guidelines and regulations of the University Hospital of Geneva (Switzerland), and the whole procedure including the experimental protocol was conducted in agreement with the ethical committee of the University Hospital of Geneva (Switzerland).

**Table 1:** Patient demographic and clinical data.

Patient	Sex	Handedness	Age (years)	Age at onset of epilepsy (years)	Aetiology	Seizure focus	Resection	Seizure frequency	Seizure type	Drugs and dose (mg/day)	Education completed	Side of implantation
3	F	L	27	14	Idiopathic/cortical atrophy L	Parietal operculum L	Left post-central and parietal opercular	Monthly	SPS	LTG 600, LVT 2000	Tertiary	L
4	F	R	53	25	Idiopathic	Temporal R	Right anterior temporal/temporal lobectomy	Weekly	SPS	CBZ 450	Secondary	R
5	M	R	21	16	Leukoencephalopathy	Temporal R	Right temporal/no resection	Weekly	SPS	VPT 2000, CBZ 1,800	Tertiary	L + R
10	M	R	34	23	Idiopathic	Temporal R	Right supero-lateral temporal/temporal lobectomy	Monthly	CPS	LVT 1,500, CBZ 800	Secondary	L + R
11	F	R	21	19	Idiopathic	Orbitofrontal R	Right orbito-frontal/ventro-polar lobectomy	Weekly	SG TCS	LTG 200, VPT 1,500, LCS 400	Secondary	R
12	F	L	26	21	Idiopathic	Insular R	Right anterior temporal/temporal lobectomy	Monthly	SG TCS	CBZ 1,800	Primary	R
13	M	R	35	19	Idiopathic	Temporal R	Left anterior temporal lobe resection with amygdalo-hippocampectomy	Monthly	SG TCS	LTG 600, ZNS 200	Tertiary	R

*CBZ* carbamazepine, *CPS *complex partial seizure, *VPT* valproate, *LCS* lacosamide, *LTG* lamotrigine, *LVT* levetiracetam, *SG TCS* secondary generalized tonic clonic seizure, *SPS* simple partial seizure, *ZND* zonisamide.

The patients had normal or corrected-to-normal vision, and no history of head trauma or encephalitis (see Table 1 for clinical details). Two patients had bilateral implants in the amygdala, one had an implant in the left amygdala, and four patients had an implant in the right amygdala. Furthermore, three amygdalae were excluded from analysis due to excessive noise in the signal, resulting in a total of nine amygdalae in the final dataset.

### Stereotaxic contacts localization

**Figure 1:**
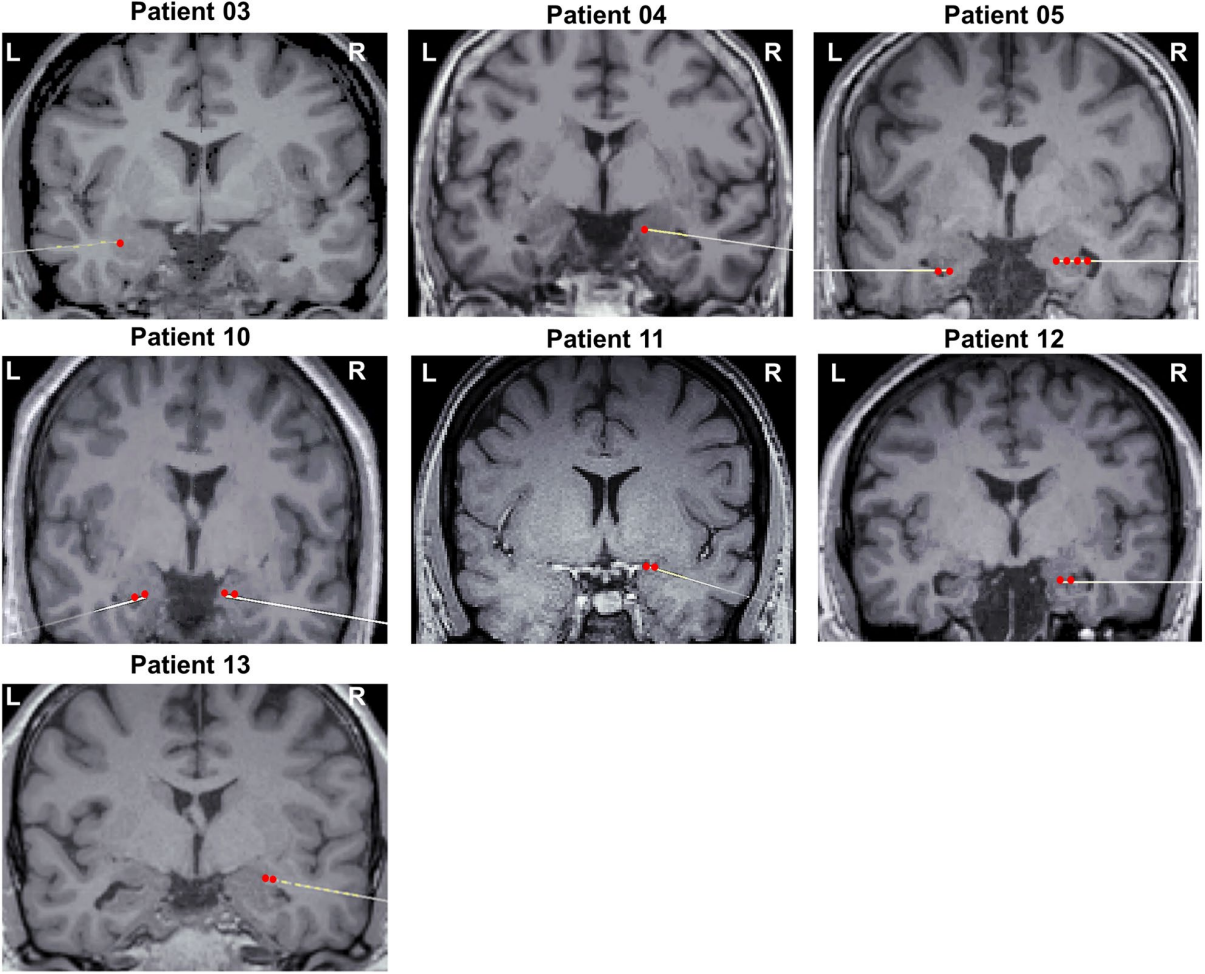
Electrode localization in the amygdala. Electrode contact localization in 9 amygdalae of 7 patients (5 left, total 21 contacts). Coronal sections of post-implant electrode insertion in individual CT-scans, coregistered with corresponding normalized MRI scans. The images illustrate radiologically normal amygdalae. Red dots indicate the contacts included in our analyses.

Electrode contact localization was done in the native structural MRI space. A CT scan was performed after implantation, which was co-registered with the T1 MRI image obtained before surgery, using the Brainstorm toolbox^39^ and custom-written scripts for Matlab (Mathworks, R2014b). Figure 1 illustrates the radiologically normal amygdalae and electrode locations for all individual patients.

### Experimental procedure

In order to evaluate the influence of task-relevance on emotional and non-emotional visual stimulus processing, we used a modified version of a Go/No Go task^40,41^ where equal probabilities of targets (Go stimuli) and non-targets (NoGo stimuli) were presented to the participants in order to avoid oddball effects^26^, and to minimize motor inhibition efforts that would take place with infrequent non-target trials, as typically observed during a classical Go/NoGo task^42^. Only Go stimuli required a motor response. Two stimulus-categories were used in two separate tasks. The first task (FACE task) included fearful and neutral faces, whereas the second task (SHAPE task) used circles and squares (see the “Stimuli” section). The full experiment included one block per stimulus, where participants were instructed to solely respond to one particular stimulus type within a category (i.e. targets) and to ignore the other stimulus type (i.e., non-targets). Each task was divided into 2 different blocks reversing which stimulus was the target. The order of blocks was counterbalanced across participants. For instance, one participant started with fearful faces as targets, whereas another participant started with neutral faces as targets in the FACE task. Stimuli were identical across the different task-relevance conditions, i.e. the same fearful face would be target in one block, but non-target in the other. Within each block, the stimuli were pseudo-randomly presented, in order to avoid presenting the same stimulus twice consecutively.

**Figure 2:**
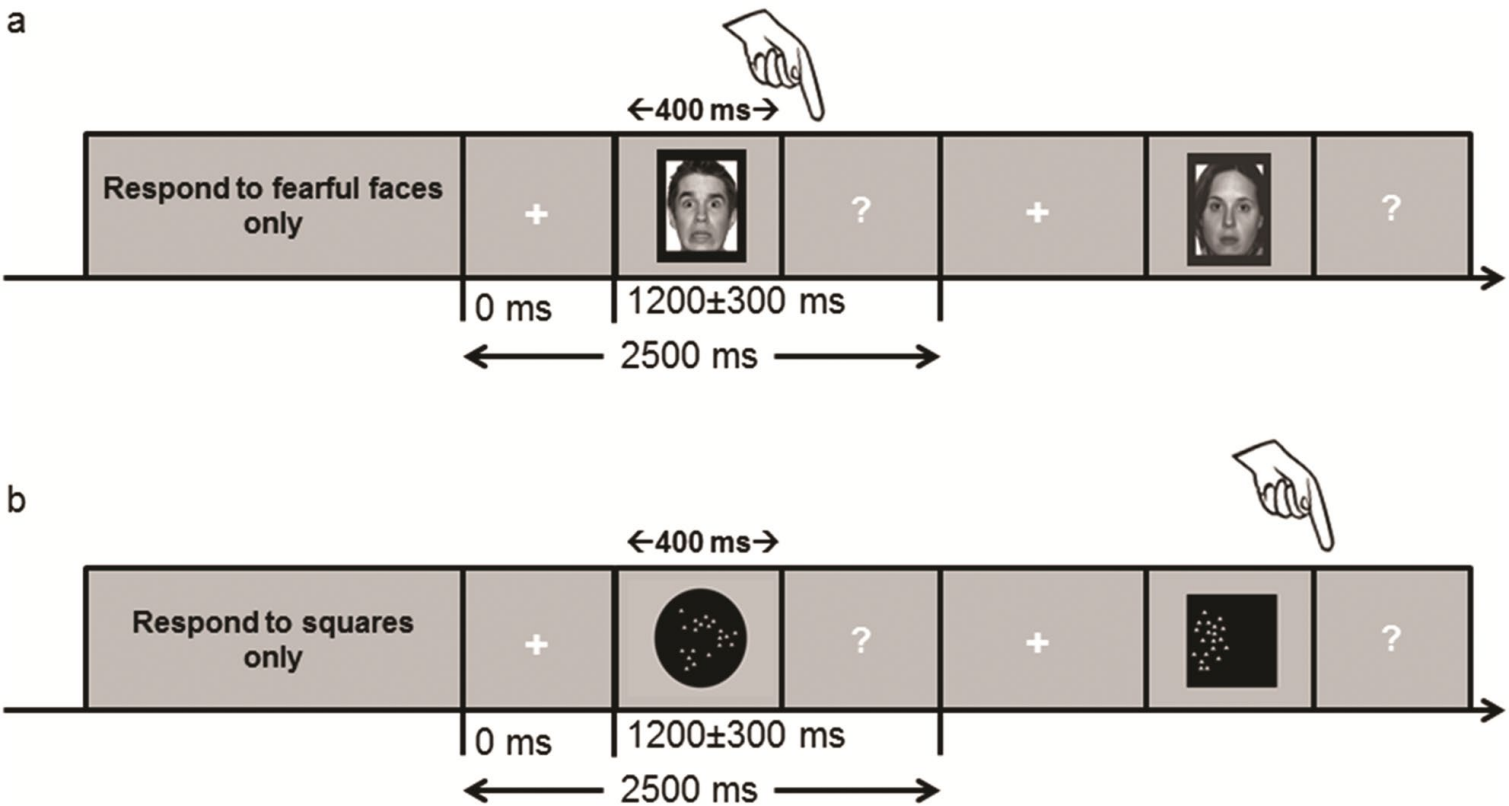
Schematic representation of the face and shape tasks. (**a**) Example of a trial in the face task where fearful faces were targets and neutral faces were non-targets. In another sub-block, neutral faces were targets and fearful faces were non-targets. (**b**) Example of a trial in the shape task, where squares were targets and circles were non-targets.

Testing was performed at bedside in a dedicated quiet room. Patients sat comfortably at ~ 60 cm distance from the screen. Prior to recording, the task was carefully explained and illustrated by a few example trials. During the experiment, each block began with a screen providing the corresponding instruction (e.g. “Respond to fearful faces only”). The sequence within each trial was composed as follows (Fig. 2). First, a white fixation cross appeared for a duration varying from 900 to 1500 ms (randomly jittered and balanced across conditions); then, the stimulus was presented for 400 ms; and finally, a white question mark was displayed. Responses were recorded during the whole interval between stimulus pictures, and all trials had a constant total duration of 2500 ms (including jitters).

The participant was asked to press a response button as soon as they detected the target stimulus type among the current visual category. Patients did not receive explicit instructions to respond as quickly as possible, but the response-window had a limited duration (as indicated above) and thus maintained a certain pressure to respond immediately. The background screen was of grey color throughout the whole experiment. In total, 112 trials were presented in each block, with short breaks between blocks if requested by the participants. Stimulus presentation was controlled with the software E-prime (Neurobehavioral Systems, USA). The latency of stimulus appearance on screen was verified offline with a photodiode, and the time delay between stimulus appearance and trigger was corrected accordingly.

### Stimuli

A set of 28 faces (50% females) taken from the Nimstim^43^ and Karolinska^44^ databases, normalized for luminance, were used in this experiment. Faces conveyed either a neutral (NEU) or a fearful (FEAR) expression. Each stimulus was previously rated for emotion and arousal by fourteen healthy participants (4 males, mean = 27 years, SD = 3, recruited among students at the University of Geneva), using a scale that ranged from 1 to 100 (where 100 represented the most aroused, the most fearful, or the most neutral expression, respectively). These ratings indicated that, relative to neutral faces, fearful faces were reliably perceived as more arousing (FEAR: mean = 70.12, SD = 4.39, NEU: mean = 26.01 and SD = 7.59, t_13 _= 50, *P* < 10^–16^), more fearful (FEAR: mean = 81.04 and SD = 7.59, NEU: mean = 8.60 and SD = 4.29, t_13 _= 130, *P* < 10^–21^), and less neutral (FEAR: mean = 5.16 and SD = 3.5, NEU: mean = 77.08 and SD = 12.97, t_13 _= 56, *P* < 10^–17^).

The geometric shapes consisted of 14 black circles and 14 black squares, custom-made with graphic software. Their size was matched so that circles and squares would have approximately the same visual surface on the screen. In order to obtain some variability across different shapes (as it is the case with faces), the circles and squares were filled with small grey triangles randomly interspersed within the shape area (see Fig. 2). This conferred the shapes some variability in individual identity, while their general layout was kept identical across stimuli and conditions (target vs. non-target). The stimuli were presented on a LCD screen and covered about 15° of visual angle.

### Data acquisition

Intracranial EEG data were acquired on a Micromed System Plus (Micromed, Italy) with a sampling rate of 2048 Hz and an online high-pass filter of 0.02 Hz. Stainless electrode arrays consisted of 8 contacts for all patients, except one, whose amygdalae were implanted with electrodes containing 10 contacts (AD-Tech, electrode diameter: 3 mm, inter-contact spacing: 2 mm). Reference was initially set to Cz prior to recording, and data were re-referenced to the nearest white matter contact of the same electrode stripe for analysis.

### Signal pre-processing

A low-pass non-causal Butterworth filter (200 Hz) and a notch filter (50, 100, 150 Hz) were applied with the Cartool software^45^. Further signal pre-processing was carried out with custom-written scripts for the toolbox Fieldtrip^46^ and implemented with Matlab (Mathworks, R2012). For the iERPs, data were detrended, epochs from − 200 to 1000 ms were then segmented and baseline corrected relative to the 100 ms prior to stimulus-onset. Then, each trial was visually inspected, and trials containing excessive noise, or as epileptic spikes, were excluded from further analysis. Trials corresponding to false alarms or omissions were also excluded from further analyses. Then, the average number of trials across amygdalae was 38.7 in each experimental condition (Supplementary Table 4), with no differences among conditions (*P* > 0.1).

For the time–frequency analysis, large epochs of − 2000 to 3000 ms were extracted from the data and a baseline correction relative to activity from − 300 to 0 ms was applied. Time–frequency data were obtained with multitapers (Fieldtrip options “mtmconvol” and “hanning”) from 2 to 200 Hz, in steps of 2 Hz and smoothing of 20 ms. Then, similar to previous report^47^, this signal was divided into three frequency bands, from 4 to 30 Hz (i.e., low frequency), 30 to 100 Hz (low gamma), and 100 to 200 Hz [high gamma (HG)]. This frequency decomposition enabled a direct comparison of different frequency bands in the time domain^48^. Finally, the very same trials were used for iERPs and time–frequency analyses.

A total of 9 amygdalae were included in the analysis. All signals were finally down-sampled to 512 Hz. Trials were averaged for each condition and for each contact. Amygdala contacts showing clear stimulus-driven response by visual inspection of grand averages (all conditions collapsed) were selected for further analysis (1–3 per electrode). Intracranial ERPs and oscillatory activity in the different frequency bands were averaged across selected contacts within each amygdala, in order to obtain one aggregate LFP value per subject and amygdala, and for each experimental condition.

### Statistics

To test for any effect of Emotion-relevance (FEAR vs. NEU) for the FACE task, Stimulus-type (CIRCLE vs. SQUARE) for the SHAPE task, or Action-relevance (target vs. non-target) in both tasks, as well as any interaction between these factors, we submitted our iERPs (voltage amplitude values over successive time-points) to two-tailed paired *t *tests using permutation analysis (see below) over a large time window of interest (from 0 to 1000 ms post stimulus onset). Main effects of Emotion-relevance, Stimulus-type, and Action-relevance were obtained by pooling the corresponding sub-categorical conditions. To test for interactions with the same permutation analysis, we used a subtraction method between pairs of conditions. For instance, for the FACE task, the Emotion-relevance x Action-relevance interaction was determined by subtracting the individual average in the FEAR-non-target conditions from FEAR-target, and then comparing this difference to the equivalent subtraction for the NEU conditions^49^.

Statistics were performed with Fieldtrip^46^ implemented with Matlab 2012b, using methodology similar to other recent studies with iEEG in humans^30,50^. Permutations were applied using the Monte Carlo method to the average amplitude values obtained at each time-point between pairs of conditions, within each factor and amygdala. After a permutation step, a paired t-test was calculated at each time point (within the whole 1000 ms-window from stimulus-onset) for each condition separately, with a cluster-threshold of *P* < 0.05. Significant clusters were defined by temporal adjacency of significant effects. For each cluster, the T-values were summed and the greatest sum among all clusters was entered into the permutation distribution. Permutation steps were repeated 1,000 times. Maximum T-values over time-points within the cluster are reported. Given that the permutation test is non-parametric, no degrees of freedom are given. Empirical t-clusters located below or above the 97.5 percentile were considered as statistical significant (and corrected—Corr—for multiple comparisons, as maximum clusters have been sampled during the permutation process). In addition, we also report uncorrected (Unc) *P* values but with a minimum of 10 consecutive milliseconds under the alpha threshold of 0.05. These uncorrected results are mainly provided for completeness, and our interpretation of results is primarily based on the corrected data. We used the same statistical pipeline for both the iERPs and the time–frequency analyses. Finally, to support our grand-average results with individual results, we also present scatterplots depicting the individual average amplitudes over significant time-windows, for each significant cluster (see Supplementary Fig. S1).

Furthermore, to complement our analyses, we conducted additional comparisons to characterize the amygdala response dynamics during the first and the second part of each task (FACE and SHAPE, respectively). This allowed us to determine whether Action-relevance responses in the amygdala varied over the course of each task, reflecting either learning or habituation processes. To address whether the Action-relevance responses in the amygdala were a learned process or rather habituated over time, we analyzed the first and second parts separately for each task (following the same methodology as described above, including 16 trials from the first and the second half), and tested for any interaction between the first and the second halves.

Finally, for behavioral analysis, response times (RTs) and accuracy were compared by paired *t *tests among conditions, following the same analysis rationale as used with the iERPs. Trials where RT exceeded by two standard deviations from the mean were excluded from this analysis.

## Results

### Behavioral performance

#### Accuracy

Patients performed the task with high accuracy. In the FACE detection tasks, they correctly responded (hit trials) to 89% (SD = 15.5) of the FEAR-target and 90% (SD = 2.1) of the NEU-target stimuli (no significant difference, t_6 _= 1.21, *P* = 0.83; see other behavioral results for each task condition in Supplementary Table 1). In the SHAPE detection tasks, patients correctly responded to 98% (SD = 1.9) of CIRCLE-target and 98% (SD = 1.4) of SQUARE-target stimuli (again, no significant difference, t_6 _= − 0.29, *P* = 0.81). Moreover, hit rates did not differ between the two tasks (FACE vs. SHAPE, t_6 _= 1.586, *P* = 0.16; see other comparisons between tasks in Supplementary Table 2). Patients committed very few false alarms, with rare incorrect responses to FEAR-non-targets (2.12%, SD = 1.26%) and NEU-non-targets (1.12%, SD = 0.74%) in the FACE detection task (no significant difference, t_6 _= 1.96, *P* > 0.09), and likewise for the SQUARE-non-targets (1.34%, SD = 1.67%) and CIRCLE-non-targets (1.34%, SD = 2.05%) in the SHAPE task (no significant difference, t_6_ < 0.01, *P* > 0.9). Moreover, false alarm rates did not differ between both tasks (FACE vs. SHAPE, t_6 _= 0.37, *P* = 0.68). Finally, patients committed very few omissions in the FACE detection task (9%, SD = 15.3 for FEAR-targets and 2%, SD = 2.2 for NEU-targets; no significant difference, t_6=_1.3, P = 0.24); and even fewer in the SHAPE task (0.7%, SD = 0.7 for CIRCLE-targets and 1%, SD = 0.9 for SQUARE-targets; no significant difference, t_6 _= 1.15, *P* = 0.47). Omission rates did not differ between both tasks (FACE vs. SHAPE, t_6 _= 1.77, *P* = 0.11). These results suggest that the Go/NoGo task was easily performed by patients, with globally similar difficulty across the different conditions and across tasks.

To test for any changes in performance during the course of the testing, we also compared accuracy between the first and the second halves of each task (FACE or SHAPE), for each experimental condition, but found no significant difference (all *P* > 0.58, see Supplementary Table 3).

#### Response time

In the FACE task, correct RTs to FEAR-target versus NEU-target stimuli did not differ (respectively, mean = 610 ms, SD = 184 ms; vs. mean = 673 ms, SD = 147 ms; t_6 _= − 1.74, *P* > 0.54). In the SHAPE task, RTs to CIRCLE-target and SQUARE-target stimuli did not differ either (respectively, mean = 507 ms, SD = 69 ms; vs. mean = 519 ms, SD = 83 ms; t_6 _= − 0.71, *P* > 0.8). However, target shapes were discriminated faster than face expressions (respectively, mean = 514 ms, SD = 73 ms; vs. mean = 658 ms, SD = 162 ms; t_6 _= 2.55, *P* < 0.037), presumably reflecting that shape categorization was a simpler visual task than emotion expression categorization. Comparisons of RTs between the first and the second halves of each task (FACE and SHAPE), for each experimental condition, showed no significant differences (see Supplementary Table 3).

### iEEG results

#### Face detection task

**Figure 3:**
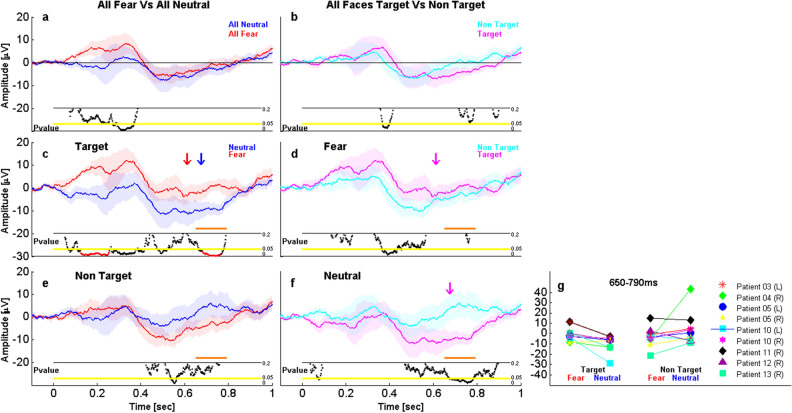
Effect of emotion-relevance and action-relevance on iERPs. (**a**) Main effect of emotion with target and non-target pooled, and (**b**) main effect of action-relevance with fearful and neutral faces pooled. Both comparisons showed no significant effects at corrected threshold. Emotional differences for target (**c**) and non-target (**e**) faces, showing significant emotion effects only for target faces from 128 to 260 ms and from 694 to 790 ms post-stimulus. Action-relevance effect for fearful (**d**) and neutral (**f**) faces, showing no significant differences. (**g**) Scatterplots depicting the individual average amplitudes for each condition, over the significant time-window where the Emotion-relevance × Action-relevance interaction was observed (650 to 790 ms). Shaded areas represent between-amygdalae standard error of the mean. The horizontal black line below the iERP waveforms indicates a *P* value threshold of 0.2 and the horizontal yellow line indicates a *P *value threshold of 0.05. Black dots below the yellow line indicate *P* values of (uncorrected) significant time-points in the comparison of both waveforms. Red dots indicate corrected results reported in the main text. Orange lines indicate significant interactions reported in the main text. Arrows above the waveforms represent the average response time for each corresponding condition (e.g., blue for neutral and red for fearful).

First, we tested for a main effect of Emotion-relevance in the FACE task, by comparing amygdala responses to FEAR faces versus NEU faces, independently of Action-relevance (interaction effects are described in detail below). For event-related potential (iERP) amplitudes, this comparison revealed differences significant at uncorrected threshold only, from 300 to 374 ms post-stimulus onset (t_8 _= 2.97, *P* = 0.021, Unc, see Fig. 3a). Likewise, we determined the main effects of Action-relevance by comparing all target faces versus all non-target faces, regardless of emotion expression, which also showed differences in iERPs from 378 to 404 ms only at uncorrected thresholds (t_8 _= 2.59, *P* = 0.032, Unc, see Fig. 3b).

Critically, there was a highly significant interaction of Emotion-relevance × Action-relevance that survived our stringent cluster correction in a late time-window from 650 to 790 ms (t_8 _= 3.15, *P* = 0.018, Corr), and that reflected distinct response patterns to the FEAR and NEU faces (Fig. 3c–f). Specifically, this interaction appeared to be driven not only by a stronger emotion effect for target faces (as opposed to non-targets, see Fig. 3c, e), but also by differential influences of Action-relevance during this late time-window (see Fig. 3d, f) with a larger negativity to target (versus non-target) faces occurring only for neutral faces. In fact, when examining the FEAR and NEU conditions separately, Action-relevance produced a late modulation for NEU stimuli (from 730 to 794 ms; t_8 _= 2.52, *P* = 0.036, Unc; Fig. 3f) but not FEAR stimuli, whereas an effect of Action-relevance was found for the FEAR faces at earlier latencies (from 376 to 430 ms; t_8 _= 2.67, *P* = 0.029, Unc; Fig. 3d).

Conversely, we also examined emotion effects for each Action-relevance condition separately. When they were targets, FEAR faces elicited an early and sustained increase in activity compared to neutral faces, peaking from 128 to 260 ms (t_8_ = 3.09, *P* = 0.015, Corr), followed by later effects from 694 to 790 ms (t_8 _= 3.62, *P* = 0.012, Corr; see Fig. 3c). Emotional effects were much less robust and delayed for non-target faces (from 554 to 572 ms, t_8 _= − 2.64, *P* = 0.03, Unc; see Fig. 3e).

Importantly, the significant interaction pattern of Emotion and Action-Relevance was generally consistent across all amygdalae from all individual patients (see individual scatterplots in Fig. 3g).

**Figure 4:**
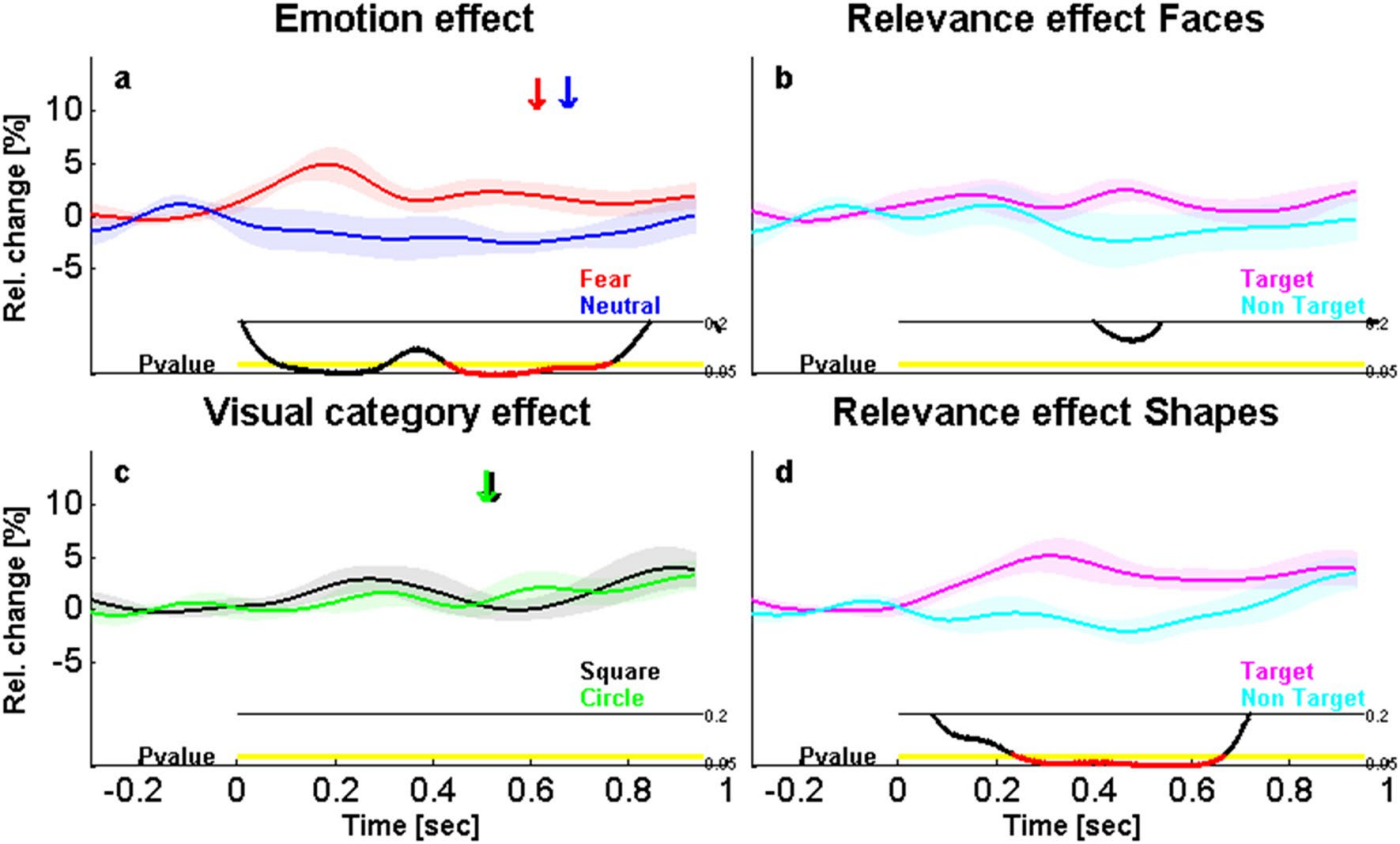
Main effect of emotion-relevance and action-relevance for both faces and shapes in High Gamma activity (HG). The activity of 9 amygdalae from 7 patients is represented. (**a**) Main effect of emotional expression for all faces (target and non-target pooled), showing significant emotion differences from 432 to 782 ms post-stimulus. (**b**) Main effect of action-relevance for faces (fearful and neutral faces pooled), showing no significant difference. (**c**) Main effect of category for all shapes (target and non-target pooled), showing no significant difference. (**d**) Main effect of action-relevance for shapes (square and circles pooled), showing significant emotion differences from 238 to 680 ms. Same color and display codes as in Fig. 3.

Besides iERPs, we performed similar analyses on the time–frequency data recorded from the same amygdala contacts in all conditions. Consistent with the above, for high gamma activity (HG), we found a significant main effect of Emotion at an uncorrected threshold from 76 to 318 ms (t_8 _= 3.11, *P* = 0.01, Unc) and then a robust difference surviving our cluster correction from 432 to 782 ms (t_8 _= 3.5, *P* = 0.006, Corr, see Fig. 4a and Fig. S1b for scatterplots depicting individual average values for significant clusters). However, there was no main effect of Action-Relevance on HG activity and no interaction. No effects were found in other frequency bands.

The fact that processing emotion from target faces elicited reliable amygdala responses, distinct from those to non-target faces, was further evidenced by comparing the iERPs of each condition to zero (baseline) activity. While response to FEAR-target faces started to differ significantly from zero at early latencies from 92 to 220 ms (t_8 _= 4.1, *P* = 0.015, Corr), response to FEAR-non-target faces differed from zero only later from 516 to 588 ms, and only when considering uncorrected results (t_8_ = − 3.06, *P* = 0.019, Unc). The same comparisons for the NEU condition demonstrated reliable differences from zero again only for target faces, from 674 to 830 ms post-onset (t_8 _= − 3.23, *P* = 0.016, Corr), but no significant difference for non-target faces. Please note however that these effects were modulated by habituation effects that took place over the course of the experiment (see further analyses below). Indeed, when considering the first and second halves of the experiment separately, we observed that response to FEAR-non-target faces also differed from zero from 100 to 218 ms in the first half of the experiment (t_8 _= 3.8, *P* = 0.003, Corr), while this difference was not significant in the second half.

In sum, both iERPs and gamma activity indicate emotion effects starting in earlier time-windows of amygdala responses to faces (before 200 ms), but interacting with distinct effects of Action-relevance at later stages of processing (after 400 ms).

#### Shape detection task

**Figure 5:**
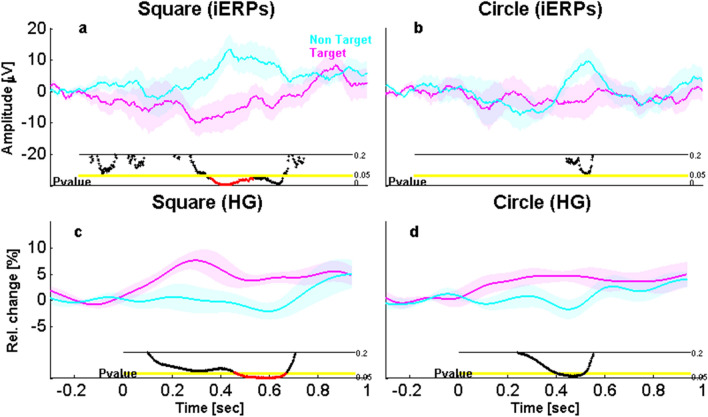
Effect of action-relevance on amygdala response to geometric shapes. (**a**) Action-relevance effect for squares (iERPs), showing a significant difference from 466 to 618 ms post-stimulus. (**b**) Action-relevance effect for circles (iERPs), showing no significant difference. (**c**) Action-relevance effect for squares (high gamma; HG), showing significant effects from 464 to 682 ms post-stimulus. (**d**) Action-relevance differences for circles (HG), showing no significant effects. Same color and display codes as in Fig. 3.

An important issue addressed by our study was whether Action-relevance is encoded in the amygdala even for non-emotional and non-social stimuli, and whether the latency of any such effect is similar or not to emotional effects observed in the FACE task. We therefore probed for a main effect of Action-relevance in the SHAPE detection task, by comparing amygdala responses to target versus non-target geometric stimuli. This comparison showed a relevance effect in iERPs from 500 to 566 ms (t_8 _= − 2.76, *P* = 0.026, Unc) and from 584 to 626 ms (t_8 _= − 2.61, *P* = 0.031, Unc) but this main effect did not survive our stringent Monte-Carlo correction threshold. However, further inspection of the data indicated that relevance effects reached corrected levels of significance in iERPs for the SQUARE condition taken alone (from 466 to 618 ms, t_8 _= 3.05, *P* = 0.021, Corr; see Fig. 5a), whereas the CIRCLE condition showed only a marginal trend (*P* = 0.06, Unc, around 600 ms post-onset; Fig. 5b).

In addition, the same comparisons in the time–frequency analysis revealed a highly significant difference in the HG band for the main effect of Action-relevance (both shapes combined) from 238 to 680 ms (t_8 _= 3.28, *P* = 0.001, Corr, see Fig. 4d). A similar pattern of activity in the HG band was also robust for the SQUARE shapes taken alone from 464 to 682 ms (t_8 _= − 3.18, *P* = 0.022, Corr; see Fig. 5c) and for CIRCLE shapes taken alone at uncorrected threshold only (from 400 to 530 ms, t_8 _= 2.67, *P* = 0.029, Unc; see Fig. 5d). No other significant results were observed in other frequency bands.

Thus, for shapes as well, amygdala responses to Action-relevance arose only during the later time window, after the early Emotion effects, in both the iERP and the time–frequency analyses.

#### Habituation and learning effects on relevance processing in the amygdala

**Figure 6:**
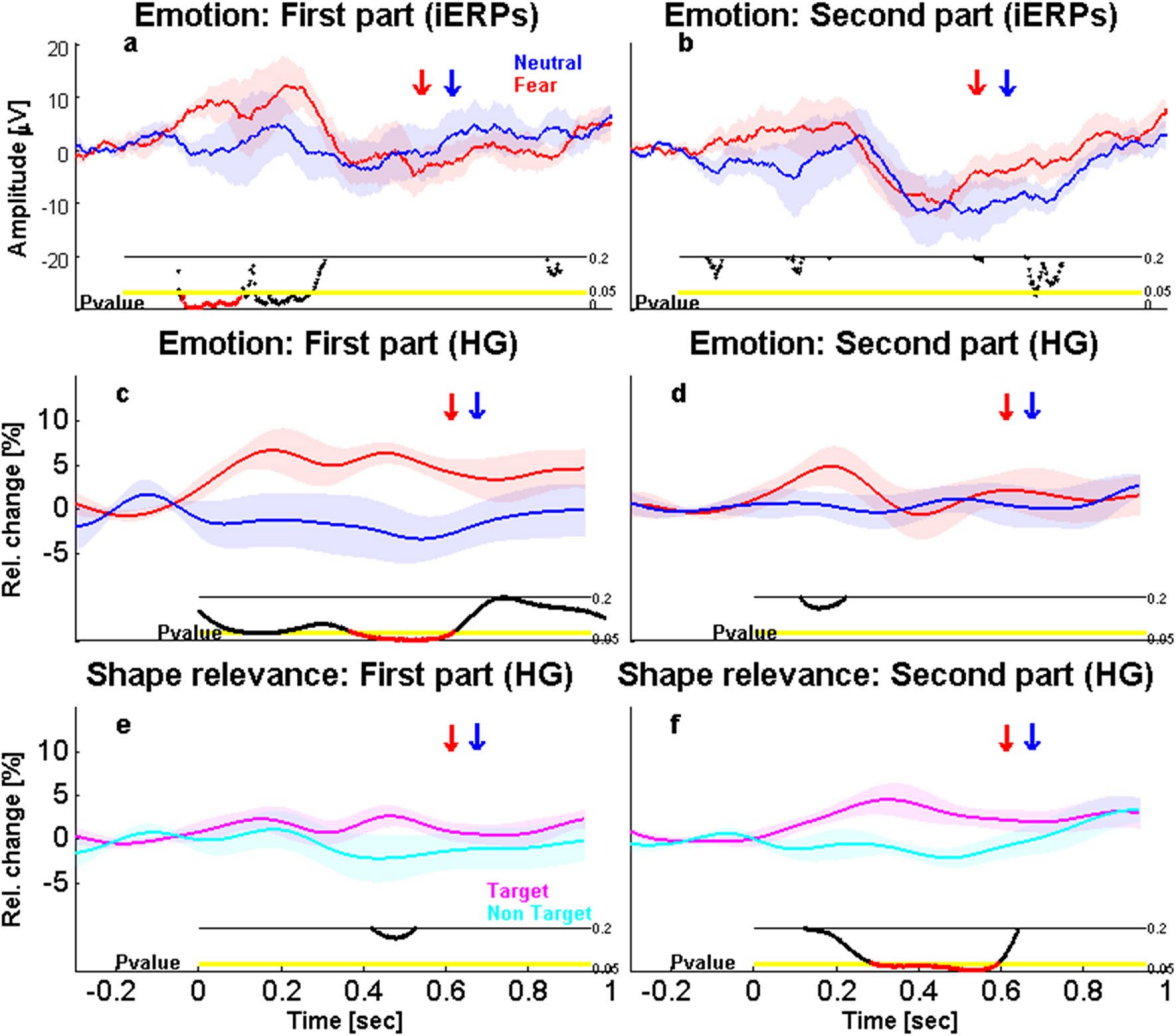
Effects of time on emotion- and action-relevance responses, across the first and the second parts of the experiment. Emotional response in iERPs during the first (**a**) and the second (**b**) part of the experiment, showing a significant effect only for the first part from 122 to 244 ms post-stimulus. Emotional response in high gamma (HG) during the first (**c**) and second (**d**) part of the experiment, showing a significant effect only for the first part from 372 to 634 ms. Action-relevance response for shapes in HG during the first (**e**) and second (**f**) part of the experiment, showing a significant effect only for the second part from 288 to 608 ms. Same color and display codes as in Fig. 3.

Given previous work describing frequent habituation of amygdala responses over successive trials^51^, we also checked for any effect of time (habituation or learning) in our study, for either type of relevance (emotion and action based). To this aim, we compared iERPs for the first and second halves of each task. In line with previous reports, we found a marked habituation for emotion in the iERPs, with strong differential responses to FEAR faces (vs. NEU) in the first half of the experiment (from 122 to 244 ms, t_8 _= 3.22, *P* = 0.01, Corr; Fig. 6a) but no significant effect in the second half (*P* > 0.05; Fig. 6b). Likewise, in HG activity, the effect of emotion was significant in the first half (from 86 to 222 ms, t_8 _= 3.22, *P* = 0.04, Unc, and, from 372 to 634 ms, t_8 _= 3.09, *P* = 0.01, Corr; Fig. 6c) but not in the second half (Fig. 6d).

In contrast, in the same task, no change was found for the Action-relevance effect on FEAR faces in either part of the experiment (all *P* > 0.05), while an opposite pattern of changes emerged in iERPs for NEU faces. Thus, Action-relevance enhanced amygdala activity to NEU faces (i.e., greater responses to target than non-target neutral faces) in the second part of the experiment (from 632 to 828 ms, t_8 _= − 3.01, *P* = 0.0009, Corr; Fig. S2b) but not in the first half (*P* > 0.2; Fig. S2a). However, no significant interaction was observed between Action-relevance (target vs. non-target) and Experimental part (first vs. second half), suggesting that although Action-relevance effects became stronger in the second half of the task, these already showed a similar trend during the first half. No other results were observed in other frequency bands.

**Figure 7:**
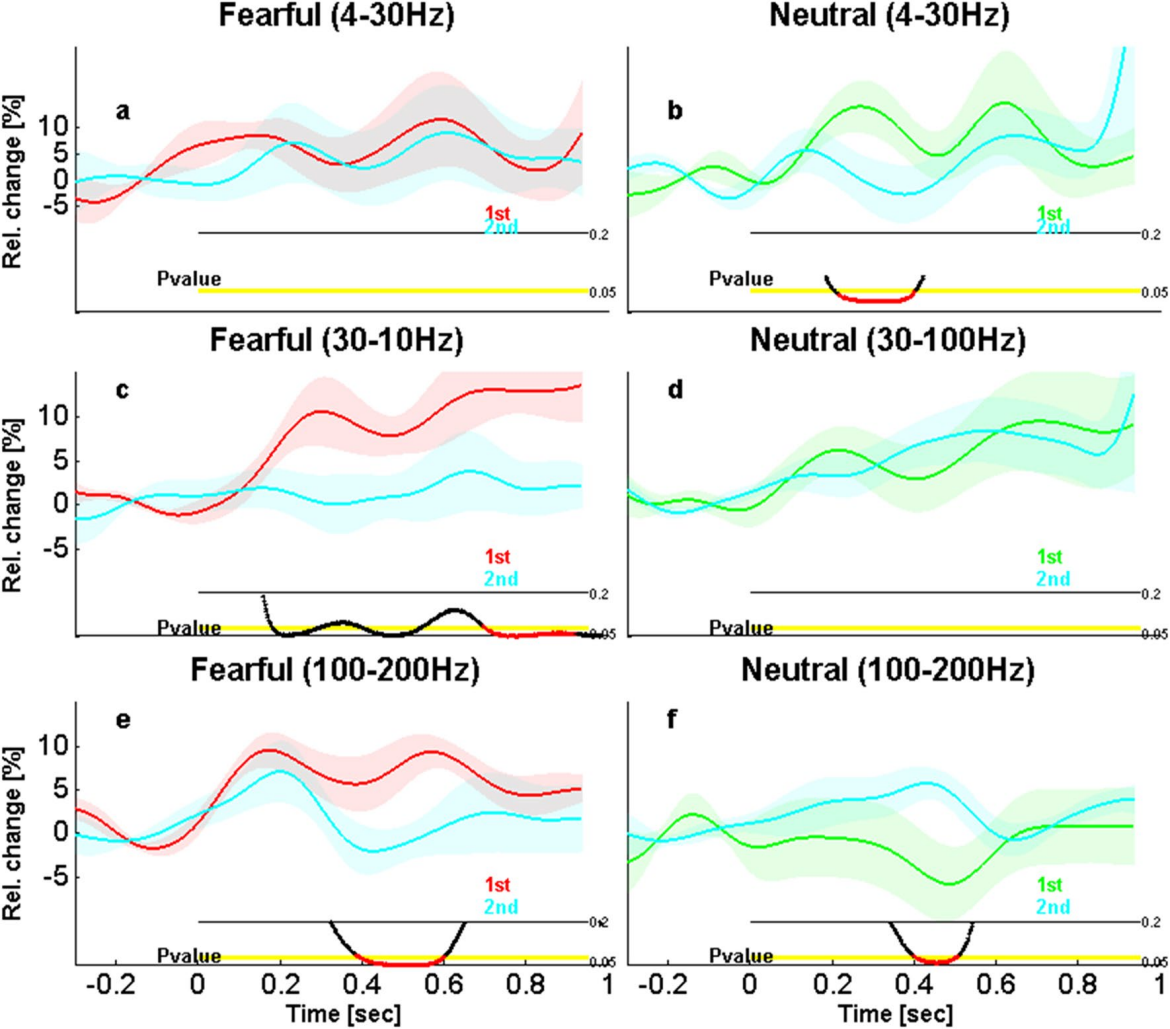
Effects of time on amygdala response in other EEG frequencies across the first and the second parts of the face experiment. (Top) Low-frequency activity (4–30 Hz) related to (**a**) fearful and (**b**) neutral non-target faces, during the first and the second part of the experiment, showing an effect only for neutral faces from 228 to 462 ms. (Middle) Low gamma activity (30–100 Hz) related to (**c**) fearful and (**d**) neutral non-target faces, during the first and the second part of the experiment, showing an effect only for fearful faces from 708 to 1000 ms. (Bottom) High gamma activity (100–200 Hz) related to (**e**) fearful and (**f**) neutral non-target faces, during the first and the second part of the experiment, showing an effect for fearful faces from 394 to 614 ms and for neutral faces from 406 to 518 ms. Same color and display codes as in Fig. 3.

To determine whether this learning effect arose from differences for the target faces or from differences for the non-target faces, we directly compared the latter conditions, for both FEAR and NEU faces. This indicated a decreased iEEG response through the experiment for all non-target faces only. A significant difference was also found between the NEU first and second halves in the low frequency band (4–30 Hz; from 228 to 462 ms, t_8 _= 3.3, *P* = 0.01, Corr; Fig. 7b), as well as between the FEAR first and second halves in the gamma band (30–100 Hz; from 184 to 316 ms, t_8 _= 2.99, *P* = 0.02, Unc, from 396 to 560 ms, t_8 _= 3.06, *P* = 0.01, Unc, and from 708 to 1000 ms, t_8 _= 3.48, *P* = 0.002, Corr; Fig. 7c). Finally, we found differences in high gamma between the first and second halves for both FEAR and NEU faces but again only for non-target faces (respectively, from 454 to 674 ms, t_8 _= 3.36, *P* = 0.02, Corr; Fig. 7e, and from 466 to 578 ms, t_8 _= − 2.79, *P* = 0.01, Corr; Fig. 7f). No other differences were found for other frequency bands, nor for iERPs, or for target faces (see Fig. S4). Together, these results suggest that only activity related to non-target faces changed between the first and the second half of the experiment.

Importantly, a similar learning effect for Action-relevance was observed in the shape task. First there was a main effect of Action-relevance in iERPs for SQUARE (targets vs. non-targets) during the second part of the experiment (from 408 to 560, t_8 _= 2.62, *P* = 0.01, Corr; Fig. S3b), but not in the first part (*P* > 0.2; Fig. S3a). No significant effect was observed for CIRCLEs in either part of the experiment. Likewise, a main effect of Action relevance was found in HG (for both shape conditions pooled) in the second half of the experiment (from 286 to 608 ms, t_8 _= 2.76, *P* = 0.01, Corr; Fig. 6f), but not in the first (see Fig. 6e). No effects were found in other frequency bands, and no differences between the first and second halves were found for target and non-target shapes, suggesting that the learning of Action-relevance for shapes was homogenous between targets and non-targets.

**Figure 8:**
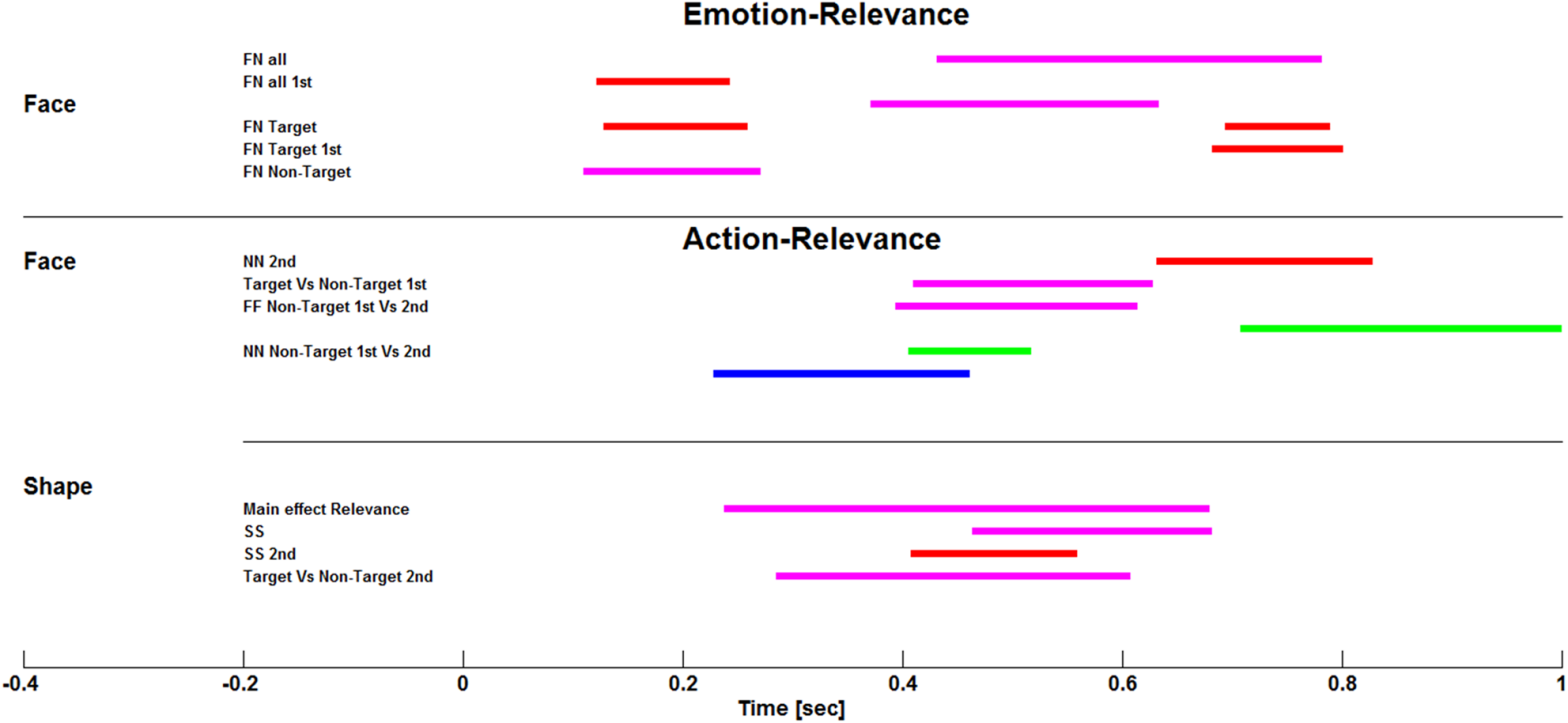
Summary of the time-course of effects observed in the amygdala in response to emotion- and action-relevance. (Top) Emotion-relevance. FN stands for fearful versus neutral; All stands for target and non-target pooled; 1st stands for the first part of the experiment. (Middle) Action-relevance for faces. NN stands for non-target neutral faces and for the second part of the experiment. (Bottom) Action-relevance for shapes. Main effect Relevance stands for targets versus non-targets for shapes, when squares and circles are considered together. SS stands for squares (targets vs. non-targets). Target versus non-target 2nd stands for squares and circles pooled together, during the second part of the experiment. Red color represents iERP findings. Blue color represents low-frequency activity (4–30 Hz) findings. Green color represents low gamma (30–100 Hz) findings. Magenta color represents high gamma (100–200 Hz) findings.

In sum, our results converge to support the idea that the encoding of emotion-relevance may generally precede that of action-relevance (see Fig. 8 for a summary), though both relevance aspects may be processed in the amygdala in an interactive manner in the later processing stages, and action-relevance processing may develop later in the course of the experiment.

## Discussion

In the present study, we aimed at identifying and comparing amygdala responses to two distinct forms of behavioral relevance, i.e., emotion-based and action/task-based. Both conditions were found to modulate amygdala activity but with different patterns and different latencies.

First, we observed in iERPs an early and robust amygdala response to emotional face expressions (relative to neutral) when these were action-relevant, arising around 128 ms post-stimulus onset. This rapid latency of amygdala responses to fear in the initial 100–150 ms time-window post-stimulus onset is consistent with emotion effects recorded from human amygdala with faces in previous iEEG studies^35^. In contrast, such an emotion response was weaker and arose at later latencies (around 550 ms post-stimulus onset) for non-target faces (which required no motor action). However, a formal statistical interaction between emotion and action-relevance was not significant for this early time-window (whereas such an interaction did emerge during later latencies, from 650 to 800 ms, reflecting differential increase to neutral face targets relative to non-targets; see below). This lack of reliable interaction between emotion- and action-relevance for the early amygdala activity may suggest that some emotional processing could occur to some degree during this time-window, regardless of the task goals, even though the direct comparison of fearful and neutral faces in the non-target condition failed to reach significance when considered alone (see Fig. 3c vs. e). Such obligatory reactivity would be consistent with fast and unintentional detection of threat cues in the amygdala to allow efficient adaptive behaviors^1, 3^, in agreement with fMRI studies reporting BOLD activation to emotional faces even when their expression is not task-relevant or presented outside the focus of attention^29,52,53^. Accordingly, a recent meta-analysis also reported consistent emotion effects in passive viewing conditions^25^, i.e., when faces are not task-relevant.

On the other hand, this non-significant interaction of emotion and action relevance might also result from low statistical power due to a small sample size. In any case, our data clearly indicate an early processing stage for emotional relevance in the amygdala, at least when also action-relevant. Hence, our iEEG results provide new insights on processing latencies for behaviorally relevant information and seem to suggest that action-related effects might act at different time-windows but are not sufficient to elicit rapid responses.

In turn, an amplification of the early emotion effects for target faces (relative to non-targets) would accord with fMRI results showing further increases in amygdala activity when emotional stimuli are targets, in comparison to non-targets^24,33^, as well as single-neuron results indicating target-specific activity in amygdala and other medial temporal lobe structures (e.g. hippocampus) during visual search^10^. Moreover, these results align with previous iEEG results where amygdala responses to emotional expression are reduced when subject’s attention is oriented to another information such as gaze serving as a cue in Posner’s task^54^. Please note that in our study all stimuli were attended and explicitly categorized in order for participants to correctly make or omit a motor response. Therefore, the effects observed here go beyond a mere influence of selective attention to the spatial location^9^ or to the emotion content of stimuli^15^, and instead more directly reflect an effect of their goal-value according to current task needs.

Alternatively, it is also possible that a weaker amygdala response to emotion expression in non-target faces might represent a motor-related inhibition of emotional arousal when stimuli are not relevant for action, an effect previously observed over prefrontal areas using scalp EEG in humans^40,41,55^. In this view, the active suppression of motor action on NoGo trials might functionally spread to stimulus representation and associated emotional processing. More broadly, these findings accord with theoretical models of emotional appraisal, whereby relevance to goals and needs (either short-term or long-term) acts as a critical context-dependent filter that shapes the processing of affective information in the amygdala^13^.

A second major finding of our study concerned amygdala reactivity to action-relevant stimuli that required an active motor response, relative to stimuli requiring no motor response, even when such stimuli were neither emotional (neutral faces) nor social (geometric shapes). Our comparison of target and non-target faces showed significant differences for neutral faces during a late window (730 ms post-stimulus), not observed for fearful faces. The absence of significant action-relevance effects for fearful faces at this latency might be explained by an overlap of a delayed negative waveform in iERPs responding to both emotion and action-relevance. In fact, a differential emotional response in this latency was also reliably observed for target faces (starting 694 ms post-stimulus), but not for non-target faces, suggesting that emotion- and task-related processes could exert a simultaneous, non-additive influence during this late time window.

In addition, our results also revealed modulatory effects of action-relevance to non-face stimuli (e.g. squares), suggesting that the amygdala may encode behavioral relevance and task-based needs even for abstract shapes, beyond the intrinsic affective value of faces and expressions. Importantly, these action-relevance effects appeared at later response latencies than emotion-relevance (i.e., starting around 220 ms and lasting until 800 ms overall). However, for both iERPs and HG, this modulation was globally weaker in comparison to emotion effects in the face task, and unexpectedly stronger for squares than for circles. This marginal (or absent) effect of action-relevance for circles may suggest that amygdala malleability to task-driven signals is sharpened or attenuated depending on stimulus similarity or association with meaningful categories (for instance with faces^56,57^), thus possibly leading to floor effects or interactive potentiation^58^. Indeed, previous work^57^ found greater amygdala activation to geometric stimuli with sharp edges compared to smooth shapes, attributed to preference biases towards a low-level perceptual properties of objects that are statistically associated with potential threat. Altogether, these observations point to the notion that goal and action-related relevance effects might be amplified by, or even contingent on, pre-existing associations acquired through prior experiences.

In keeping with this, we also found that amygdala responses to action-relevant targets increased over the course of the experiment (being larger in the second than in the first half of trials), in marked contrast with the habituation observed for emotional responses^39,40^. This pattern suggests that the processing of action relevance may be acquired and consolidated during performance of the task, according to current goal settings and response monitoring^59^. This is unlike the “intrinsic” overlearned emotional value of facial expressions that could be recognized from the outset, but then habituated with repetition. In contrast, a decrease in amygdala responses from the first to the second part of the experiment arose for non-target faces, unlike for target faces. Moreover, such changes were observed around 220 ms for neutral faces, and slightly later around 400 ms for fearful faces, suggesting a stimulus-specific adaptation time-course, consistent with the notion that amygdala response to threatening stimuli is more efficient, at least during the initial part of processing^31^. For the shape task, no such difference was observed for target or non-target stimuli, suggesting that this learning effect may be more homogenous than for faces. Together, these results indicate that amygdala activity may encode both emotion- and action-relevant events.

Overall, a role of action and goal relevance in driving amygdala activity might accord with previous work highlighting other broader appraisal effects, including a role in processing arousing information^60,61^ and in determining readiness to respond to salient inputs^62,63^, not necessarily linked to emotional (positive or negative) valence. We surmise that an acquisition of goal-dependent action values in the amygdala might be governed by top-down signals from higher-level cortices, for example in prefrontal regions that are implicated in task settings and executive attention control^64^. Top-down or feedback signals might also originate from neuromodulatory systems such as the locus coeruleus, which has been shown to be recruited during goal-directed movement^65^ with differential neuronal firing for Go but not NoGo trials^66^. As the locus coeruleus holds bidirectional connections with the amygdala, it could project back to the amygdala to enhance its reactivity to action-relevant cues^67^. More generally, an implication of top-down versus bottom-up signals onto the amygdala in order to gate the processing of action-relevant versus emotion-relevant information might explain later latencies of differential neural responses in the former condition.

### Limitations

Even though it provides important and novel insights, our iEEG study is not without limitations. First of all, we examined a total of nine amygdala obtained in epileptic patients undergoing neurosurgery. While this number constitutes a small sample limiting statistical power, it reflects the difficulty of recruiting and testing such patients in experimental paradigms. This sample size is fully in the range of past studies on emotional perception using iEEG (see for a review^68^). Nevertheless, this limitation prevents us from reaching firm conclusions regarding some of our iERP results that were marginally significant (uncorrected level) and regarding behavioral results that were statistically non-significant, given that a null effect does not equate an absence of effect. In the same line, the fact that participants were epileptic patients may be a possible cofound, limiting generalization to normal brain function, as in most other iEEG studies. This may also explain the relatively high variability among patients in their ability to discriminate the emotional expression of target faces, in line with subtle face processing deficits in temporal lobe epilepsy^69^. Nevertheless, our behavioral results indicated that our patients performed the current tasks with high accuracy, similarly to healthy subjects. Further, we note that the geometrical shapes were easier to discriminate than face expressions, as suggested by the response time results, precluding a direct comparison between our two tasks. However, this was not the main goal of our study, as the SHAPE task primarily aimed a probing “pure” action-relevance effects without any strong pre-existing stimulus significance.

Another limitation of the current data also lies in the fact that we considered the amygdala as whole, despite some contacts being more anterior and others more posterior. Thus, we were not able to dissect the role of different subnuclei that are known to compose this small subcortical structure and hold distinct populations of neurons with different neuronal response profiles^70^. Future studies might refine these findings by using single-cell recordings with micro-electrodes (not used for clinical recordings). Likewise, our sample size precluded any analysis of possible hemispheric lateralization effects^25^. On the other hand, the significant effects and dissociations observed here clear provide novel support to the hypothesis that the human amygdala may be part of a “relevance detection” system, rather than merely being a threat or emotion detector^4^, and further highlight for the first time a distinctive temporal latency for processing emotion-related and task-related information within this brain region.

## Conclusion

In sum, our results reveal not only that action-relevance can be encoded in amygdala activity, but also that different domains of relevance may be processed in this region at different time-windows depending on stimulus type or task needs (see Fig. 8 for a summary). These results extend previous results showing the implication of the amygdala in the motivational significance of motor actions with non-emotional stimuli^59^ in addition to emotional information^30,35^. A better understanding of the role of the human amygdala in coding both affective and non-affective relevance may help to shed light on disturbances associated with maladaptive reactivity and psychopathology.

## Supplementary information

Supplementary information

## Data Availability

The datasets analyzed during the current study are available from the corresponding author on reasonable request.
