# Supplementary material for: Temporal dynamics of amygdala response to emotion- and action-relevance
Source: Sci Rep. 2020 Jul 7;10:11138. doi: 10.1038/s41598-020-67862-1 (PMC7340782; doi:10.1038/s41598-020-67862-1)
Supplement: Supplementary file 1 — Supplementary information [file 41598_2020_67862_MOESM1_ESM.pdf]

Title: "Temporal dynamics of amygdala response to emotion- and action-relevance"

Running title: "Amygdala relevance processing"

Author names and affiliations: Raphael Guex[1,2,3,4*], Constantino Méndez-Bértolo[5], Stephan Moratti[6], Bryan A. Strange[7,8], Laurent Spinelli[2], Ryan J. Murray[3,9], David Sander[3,9], Margitta Seeck[2], Patrik Vuilleumier[1,3] & Judith Domínguez-Borràs[1,3,4,†]

[1]Laboratory for Behavioral Neurology and Imaging of Cognition, Campus Biotech, University of Geneva, Geneva, Switzerland, [2]Pre-surgical Epilepsy Evaluation Unit, Clinic of Neurology, University Hospital, Geneva, Switzerland [3]Swiss Center for Affective Sciences, University of Geneva, Geneva, Switzerland, [4]Department of Clinical Neurosciences, University of Geneva, Geneva, Switzerland, [5]Facultad de Psicología, Universidad Autónoma de Madrid, Spain, [6]Department of Experimental Psychology, Complutense University of Madrid, Spain, [7] Laboratory for Clinical Neuroscience, Centre for Biomedical Technology, Universidad Politécnica de Madrid, Madrid, Spain, [8]Department of Neuroimaging, Alzheimer's Disease Research Centre, Reina Sofia-CIEN Foundation, Madrid, Spain, [9]Laboratory for the Study of Emotion Elicitation and Expression, Department of Psychology, University of Geneva, Geneva, Switzerland.

[†]Current affiliation: Department of Clinical Psychology and Psychobiology, University of Barcelona, Spain.

***Corresponding author:** Raphael Guex. Laboratory for Behavioral Neurology and Imaging of Cognition, Department of Neuroscience, University Medical Center, 1 rue Michel-Servet, CH-1211 Geneva, Switzerland, Tel: +41223790347. Fax: +41223795402, email: raphaelguex@yahoo.fr

Email addresses: raphaelguex@yahoo.fr, cmendezbertolo@gmail.com, smoratti@psi.ucm.es, bryan.strange@upm.es, Laurent.spinelli@hcuge.ch, ryan.murray@unige.ch, david.sander@unige.ch, Margitta.Seeck@hcuge.ch, Patrik.vuilleumier@unige.ch, Judith.dominguezborras@unige.ch

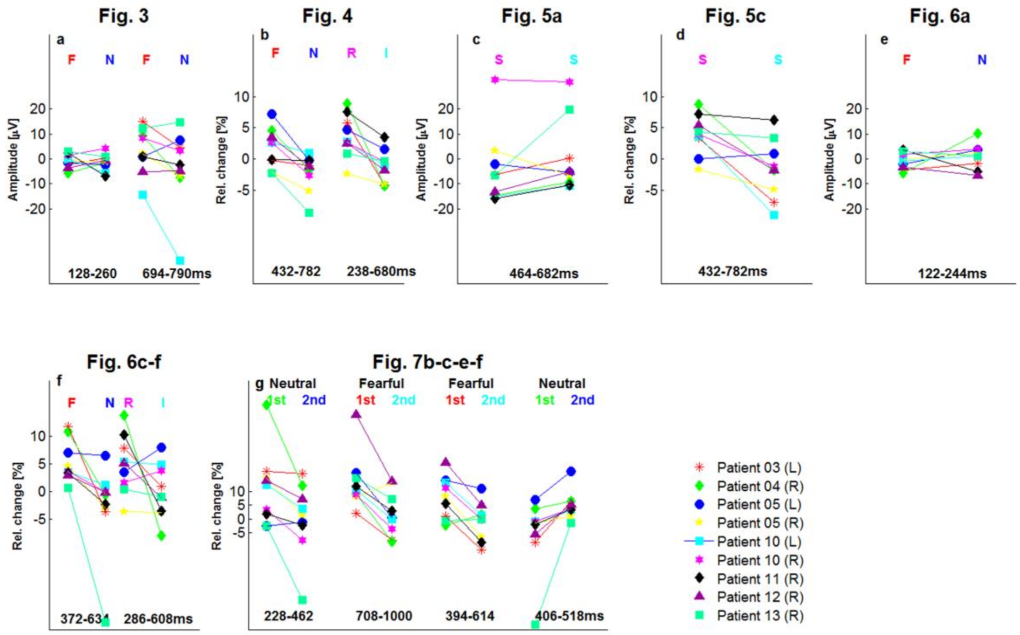

Figure 1 Supplementary. Scatterplots depicting the individual average amplitudes for each condition, over the significant time-window of each effect reported. *(a)* ***Fig. 3c***. *(b)* ***Fig. 4ad***. *(c)* ***Fig. 5a***. *(d)* ***Fig. 5c***. *(e)* ***Fig. 6a***. *(f)* ***Fig. 6c,f***. *(g)* ***Fig. 7bcef***. *F stands for fearful and N for neutral faces; R stands for action-relevant (target) and I for action-irrelevant (non-target). S stands for square and C for circle.* 1st *stands for first part of the experiment and* 2nd *for the second part. Same color and display codes as in* ***Fig 5***. *See corresponding figures for more information.*

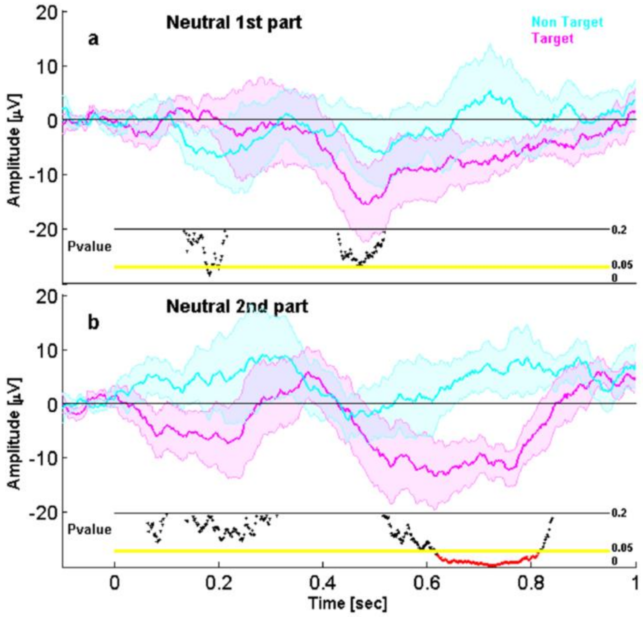

Figure 2 Supplementary. Action-relevance learning effect for neutral faces. *(a) Action-relevance differences for neutral faces during the first part of the FACE task, showing no significant effects. (b) Same differences during the second part of the FACE task, showing significant action-relevance effects from 632 to 828ms post-stimulus. Same color and display codes as in* ***Fig 3****.*

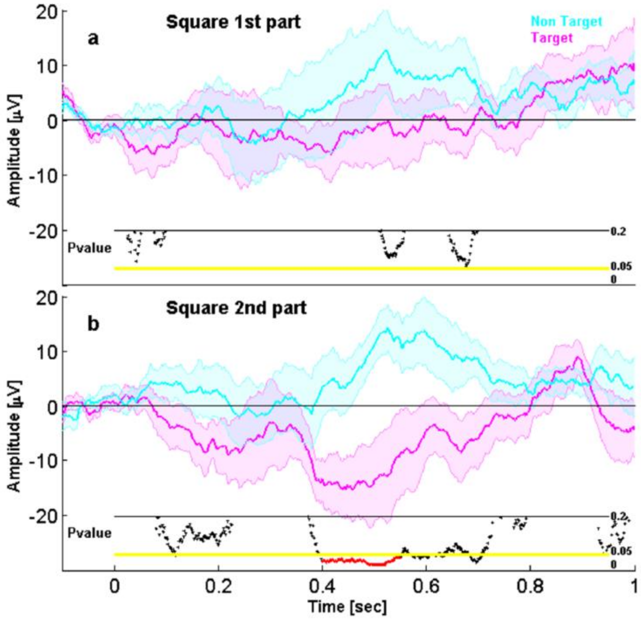

Figure 3 Supplementary. Action-relevance learning effect for squares. *(a) Action-relevance differences for squares during the first part of the SHAPE task, showing no significant effects. (b) Same differences during the second part of the SHAPE task, showing significant action-relevance effects from 408 to 560ms post-stimulus. Same color and display codes as in* ***Fig 3****.*

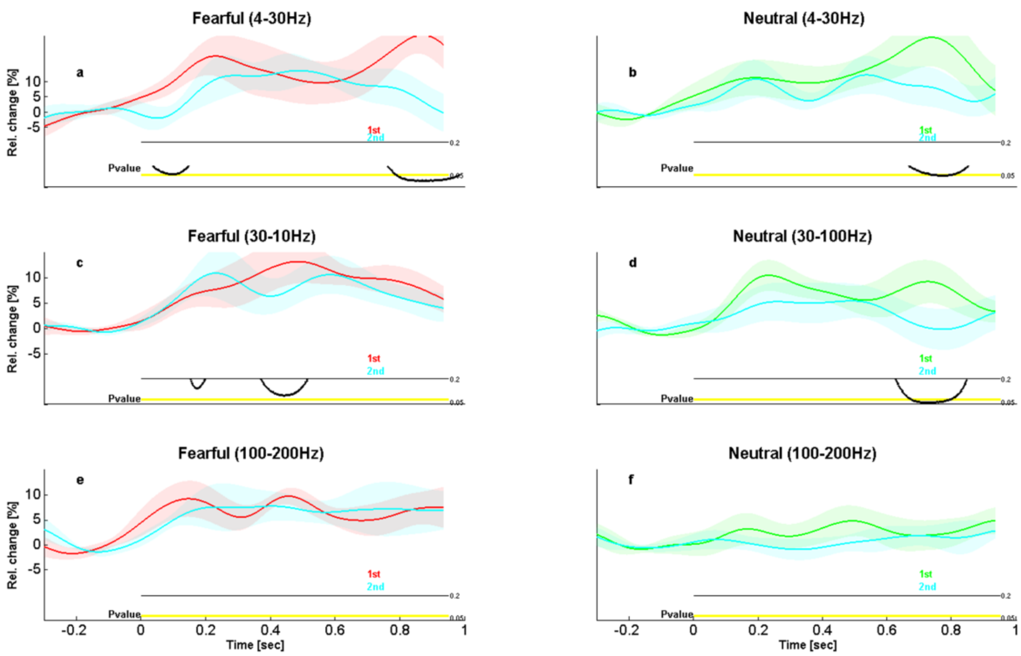

Figure 4 Supplementary. Processing of target faces in the amygdala across the first and the second part of the experiment. *(Top) Low frequency activity (4-30Hz) related to (a) fearful and (b) neutral non-target faces, during the first and second parts of the experiment. (Middle) Low gamma activity (30-100Hz) related to (c) fearful and (d) neutral non-target faces, during the first and the second parts of the experiment. (Bottom) High gamma activity (100-200Hz) related to (e) fearful and (f) neutral non-target faces, during the first and the second part of the experiment. No statistically significant results (all p values below 0.05 uncorrected). Same color and display codes as in* ***Fig 3****.*

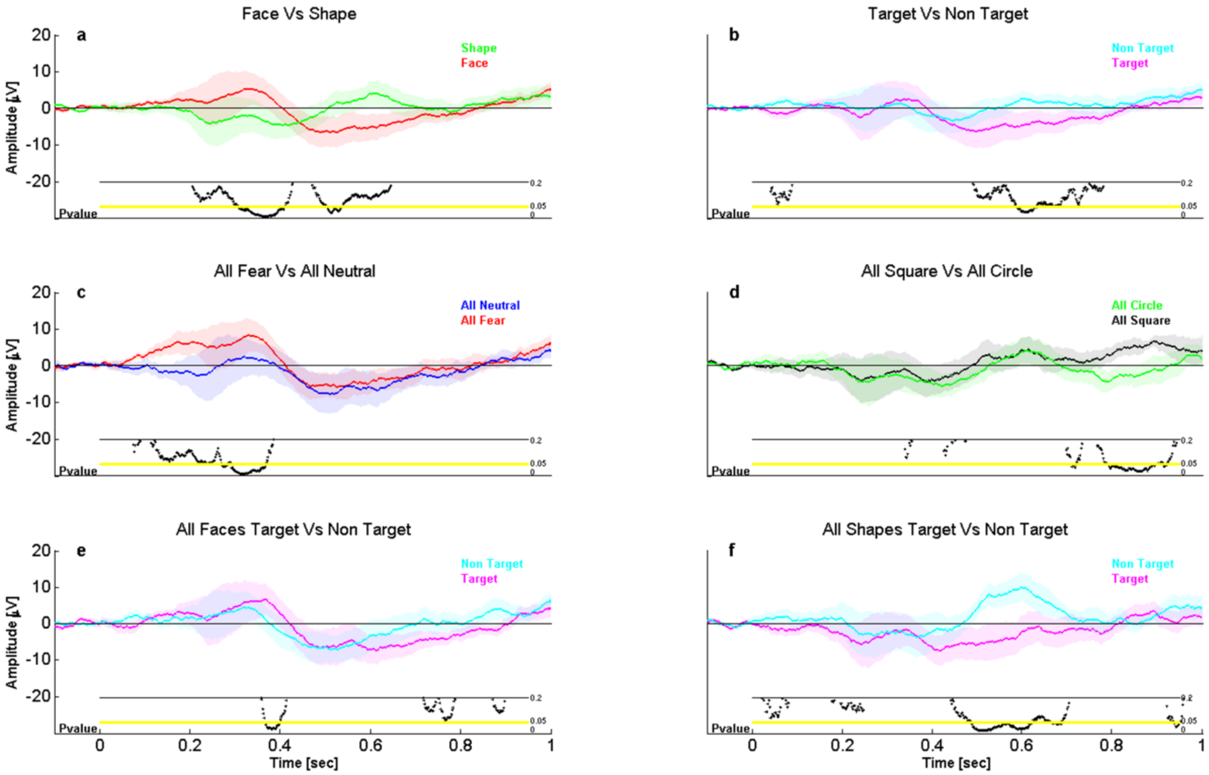

Figure 5 Supplementary. Main effects on iERPs. (a) Main task effect (all faces pooled vs all shapes pooled). (b) Main action-relevance effect (all target stimuli pooled vs all non-target stimuli pooled). (c) Main effect of emotion (all fearful faces pooled vs all neutral faces pooled). (d) Main effect of shape (all squares pooled vs all circles pooled). (f) Main action-relevance effect for faces (all target faces pooled vs all non-target faces pooled). (g) Main action-relevance effect for shapes (all shapes target pooled vs all shapes non-target pooled). Same color and display codes as in **Fig 3**.

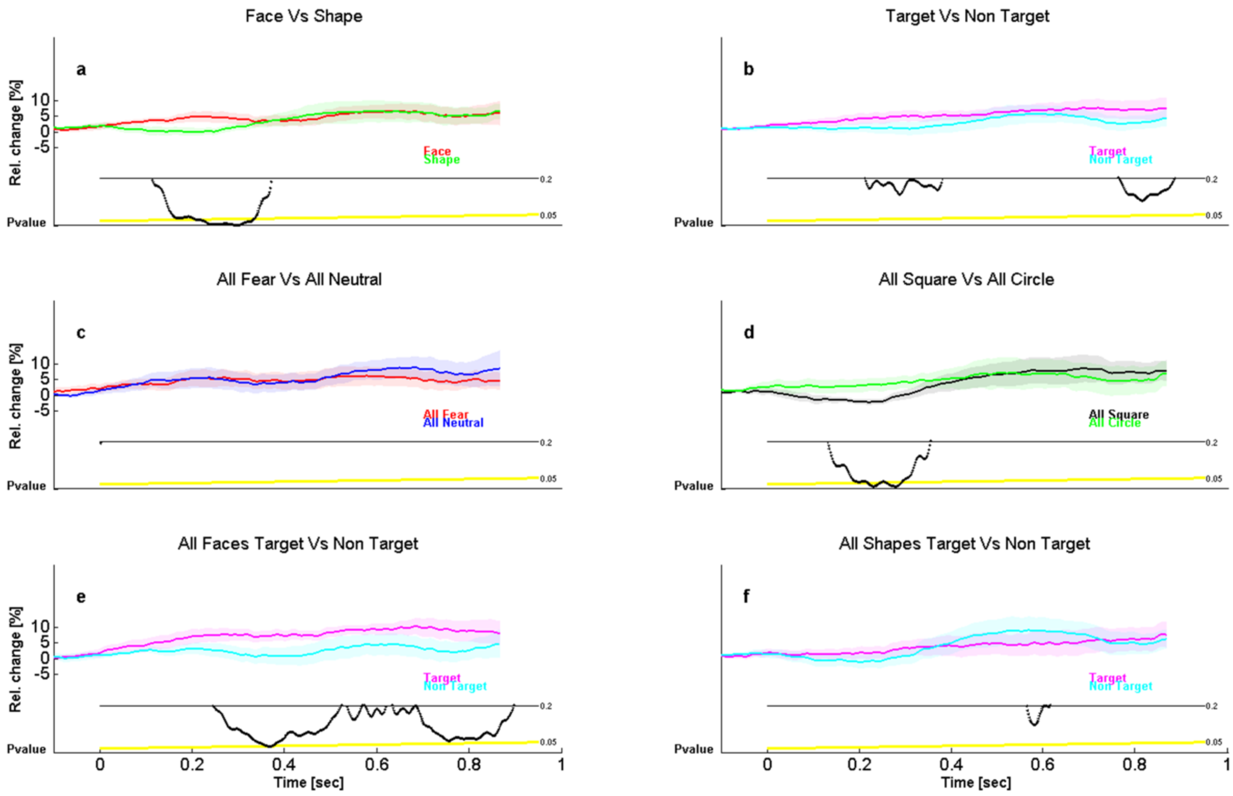

Figure 6 Supplementary. Main effect low frequencies (4-30Hz). *Same legend than Fig. S2. Same color and display codes as in* ***Fig 3****.*

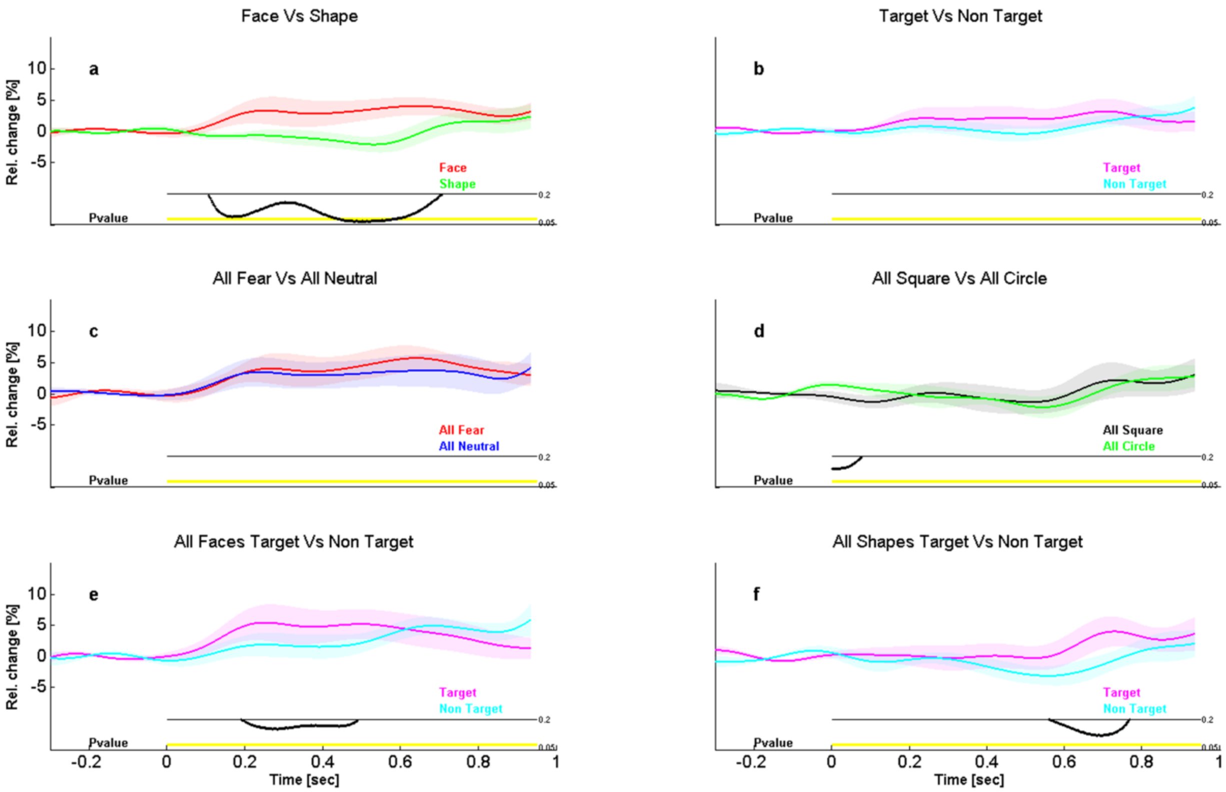

Figure 7 Supplementary. Main effect low gamma (30-100Hz). *Same legend than Fig. S2. Same color and display codes as in* ***Fig 3****.*

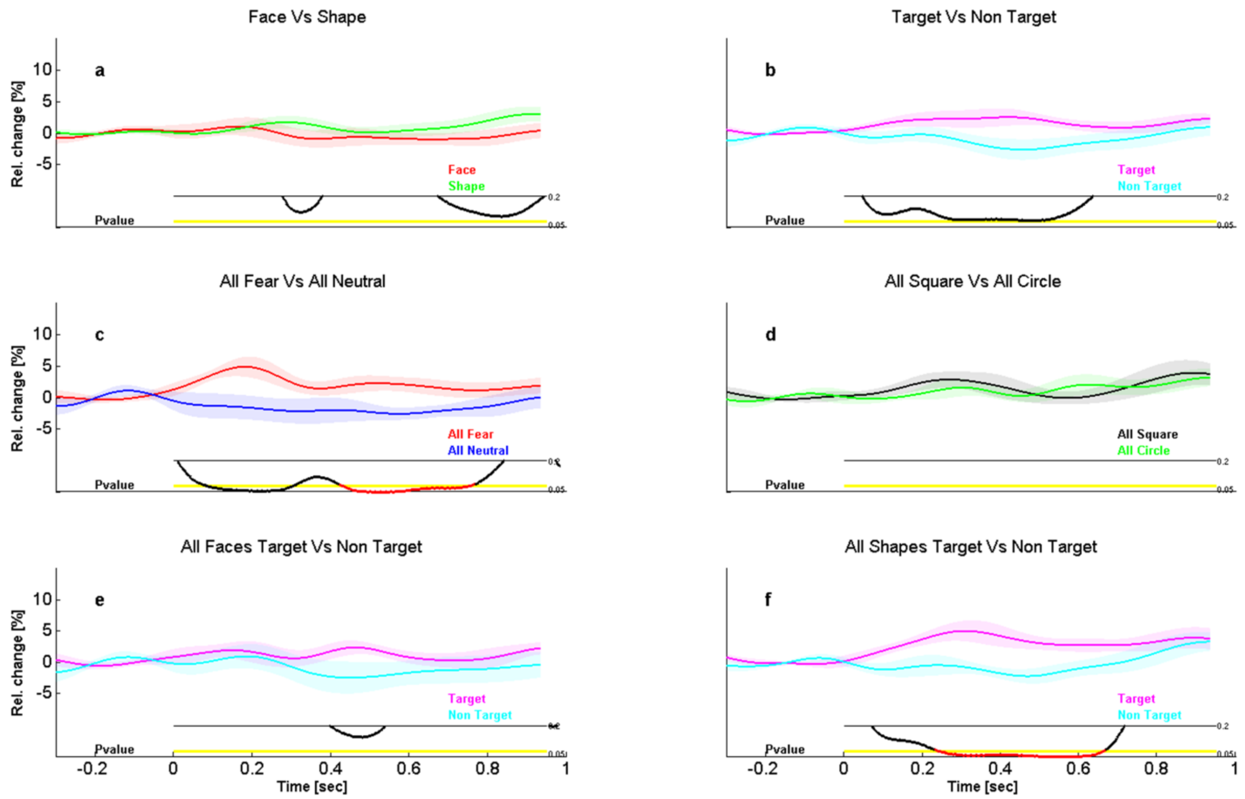

Figure 8 Supplementary. Main effect high gamma (100-200Hz). *Same legend than Fig. S2. Same color and display codes as in* ***Fig 3****.*

Hits		Statistic	False alarms		Statistic	Omissions		Statistic
Fear	Neutral	Pvalue	Fear	Neutral	Pvalue	Fear	Neutral	Pvalue
89±15.5%	90±2.1%	0.83	2.1±1.3%	1.1±0.7%	0.09	9.6±15.3%	2.6±2.2%	0.24
Square	Circle	Pvalue	Square	Circle	Pvalue	Square	Circle	Pvalue
98±1.4%	98±1.9%	0.81	1.3±2%	1.3±1.7%	1	0.7±0.7%	1±0.9%	0.47

Table 1 Supplementary. Hits, false alarms and omissions for each experimental condition, and paired t-tests across conditions of the same task. *No differences between conditions, all p >0.09.*

	Hits	Statistic	False alarms	Statistic	Omissions	Statistic
**Faces**	89.4±15%	Pvalue	1.6±0.4%	Pvalue	6±8.2%	Pvalue
**Shapes**	98.1±1%	0.16	1.3±1.6%	0.68	0.8±0.7%	0.11

Table 2 Supplementary. Hits, false alarms and omissions for faces and shapes, and paired t-tests across conditions of the two tasks. *No differences between conditions, all p >0.11.*

		Fear target	Neutral target	Square target	Circle target
**Accuracy in % and SD**	**1st half**	89±8.32	90±8.13	98±0.86	98±0.89
	**2nd half**	89±7.35	90±8.26	98±0.99	98±0.99
**Response Time in ms and SD**	**1st half**	608±174	681±168	506±65	500±79
	**2nd half**	611±140	668±135	533±109	514±68

Table 3 Supplementary. Accuracy and RT during first and second part of the experiment for each experimental condition. *No differences between conditions, all p >0.58.*

	Number Trials and SD
Fear target	38±18
Fear non-target	42±13
Neutral target	43±16
Neutral non-target	45±16
Square target	35±7
Square non-target	33±6
Circle target	37±4
Circle non-target	37±2

Table 4 Supplementary. Number of trials per condition. *(no differences between conditions, all p >0.1)*
